# Not to Miss: Intronic Variants, Treatment, and Review of the Phenotypic Spectrum in *VPS13D*-Related Disorder

**DOI:** 10.3390/ijms24031874

**Published:** 2023-01-18

**Authors:** Martje G. Pauly, Norbert Brüggemann, Stephanie Efthymiou, Anne Grözinger, Sokhna Haissatou Diaw, Viorica Chelban, Valentina Turchetti, Barbara Vona, Vera Tadic, Henry Houlden, Alexander Münchau, Katja Lohmann

**Affiliations:** 1 Institute of Neurogenetics, University of Lübeck, 23562 Lübeck, Germany; 2 Department of Neurology, University Hospital Schleswig Holstein, 23562 Lübeck, Germany; 3 Institute of Systems Motor Science, University of Lübeck, 23562 Lübeck, Germany; 4 Department of Neuromuscular Disease, UCL Queen Square Institute of Neurology, London WC1N 3BG, UK; 5 Institute for Auditory Neuroscience and InnerEarLab, University Medical Center Göttingen, 37075 Göttingen, Germany; 6 Institute of Human Genetics, University Medical Center Göttingen, 37073 Göttingen, Germany

**Keywords:** VPS13D, spastic ataxia, deep brain stimulation

## Abstract

*VPS13D* is one of four human homologs of the *vacuolar sorting protein 13* gene (*VPS13*). Biallelic pathogenic variants in the gene are associated with spastic ataxia or spastic paraplegia. Here, we report two patients with intronic pathogenic variants: one patient with early onset severe spastic ataxia and debilitating tremor, which is compound-heterozygous for a canonical (NM_018156.4: c.2237−1G > A) and a non-canonical (NM_018156.4: c.941+3G>A) splice site variant. The second patient carries the same non-canonical splice site variant in the homozygous state and is affected by late-onset spastic paraplegia. We confirmed altered splicing as a result of the intronic variants and demonstrated disturbed mitochondrial integrity. Notably, tremor in the first patient improved significantly by bilateral deep brain stimulation (DBS) in the ventralis intermedius (VIM) nucleus of the thalamus. We also conducted a literature review and summarized the phenotypical spectrum of reported *VPS13D*-related disorders. Our study underscores that looking for mutations outside the canonical splice sites is important not to miss a genetic diagnosis, especially in disorders with a highly heterogeneous presentation without specific red flags.

## 1. Introduction

There are four human homologs of the *vacuolar sorting protein 13* gene (*VPS13*), which encodes for lipid transport proteins [1]. In all homologs, pathogenic variants have been linked to different neurological disorders [2]: biallelic variants in *VPS13A* cause chorea-acanthocytosis [3] (OMIM: #200150), in *VPS13B* Cohen syndrome [4] (OMIM: #216550), in *VPS13C* early-onset parkinsonism [5] (OMIM: # 616840), and in *VPS13D,* as recently shown, spastic ataxia or spastic paraplegia [6,7] (OMIM: # 607317). However, with 31 published cases [6,7,8,9,10,11,12] of *VPS13D*-related disorder this form of *VPS13*-related disorders may be underreported.

Patients with *VPS13D*-related disorder mostly present with an ataxic or spastic gait disorder [6] with variable age at onset from infantile to adulthood onset. In addition, a variety of other signs including neuropathy (*n* = 10 [6,9,11,12]) dystonia (*n* = 7 [6,7,8]), myoclonus (*n* = 5 [6]) and chorea (*n* = 4 [7]), have been reported. There has been no identification of a clear genotype-phenotype correlation with different clinical presentations in one family being reported [9]. However, patients with severe phenotypes nearly always have one severe variant (e.g., truncating variant) and one milder variant (e.g., missense) [6]. While intronic variants have been reported in combination with truncating or missense variants, splice site effects have not yet been delineated [6].

With respect to treatment options for *VPS13D*-related disorder, there are no reports of successful treatment apart from rehabilitation therapy [11] available.

Here, we describe the first two patients with biallelic intronic variants (compound heterozygous and homozygous, respectively) and show the damaging effect of these variants in functional studies. In addition, we demonstrate deep brain stimulation (DBS) as a symptomatic treatment option for refractory tremors in one of these patients.

## 2. Results

### 2.1. Case Report: Patient 1

**Figure 1:**
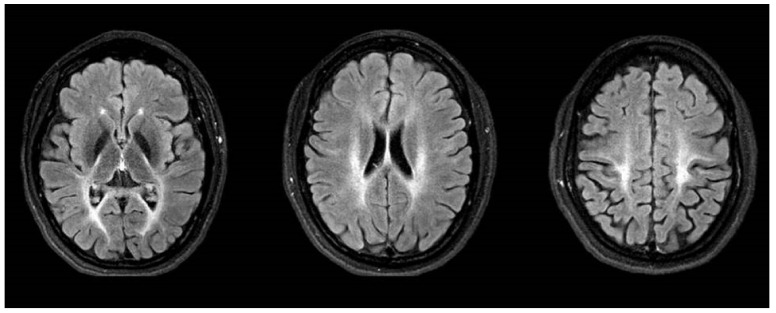
Cranial MRI in FLAIR sequence at the ages of 30 years showing severe supra- und infratentorial leukoencephalopathy with hypointense pallidum.

The now 32-year-old female patient (P1) was born after unremarkable pregnancy as the first child of non-consanguineous German parents. Cognitive and motor development was normal. At the age of 6 years, an unsteady gait was noticed. She developed pointed feet, and spastic paraplegia was suspected at the age of 8 years. The symptoms slowly progressed and at age 17, she was only ambulant with significant help and started to use a wheelchair. At that age, she also developed a tremor and ataxia in the upper extremities which progressed over the years, spreading to the head, trunk and legs and significantly affecting daily activities, e.g., preventing from independent intake of food and beverages. She also developed prominent no-no head tremor in combination with a dystonic laterocollis. Repeated cranial MRIs at the ages of 23 to 30 revealed non-progressive severe supra- und infratentorial leukoencephalopathy. The pallidum appeared FLAIR- and T2-hypointense (T2 not shown) without clear abnormalities upon iron-sensitive sequences (not shown). (Figure 1).

On clinical examination, she had severe dysarthria with scanning and voice tremor rendering her speech almost incomprehensible. She had impaired smooth pursuit, macro-saccadic intrusions as well as nystagmus while looking up and down. She had severe spastic paraplegia with increased reflexes, bilateral positive Babinski sign, and sustained foot clonus. There was mild rest, but severe slow and coarse postural and intention tremor, which was most extensive when holding out the arms in front and during finger-nose testing with an amplitude exceeding 30 cm qualifying for Holmes-like tremor. A video of the patient can be found in the Supplementary Materials (Video S1).

The patients noticed alcohol responsiveness of her symptoms, particularly the tremor, with a severe worsening of symptoms after the wearing off of the effect.

Treatment with levodopa (up to 1400 mg daily) was established at the age of 27 years leading to mild improvement of symptoms. Three years later, the patient underwent bilateral DBS in the ventralis intermedius (VIM) nucleus of the thalamus, which improved the tremor considerably enabling the patient to eat and drink independently (Video S2). The Fahn tremor scale improved from 87/144 to 70/144 immediately after the operation. Over the course of two years, however, the tremor has moderately progressed although the amplitude is still clearly below the preoperative level.

The family history was unremarkable concerning neurological disorders. The patient has one younger sister, who is healthy.

**Figure 2:**
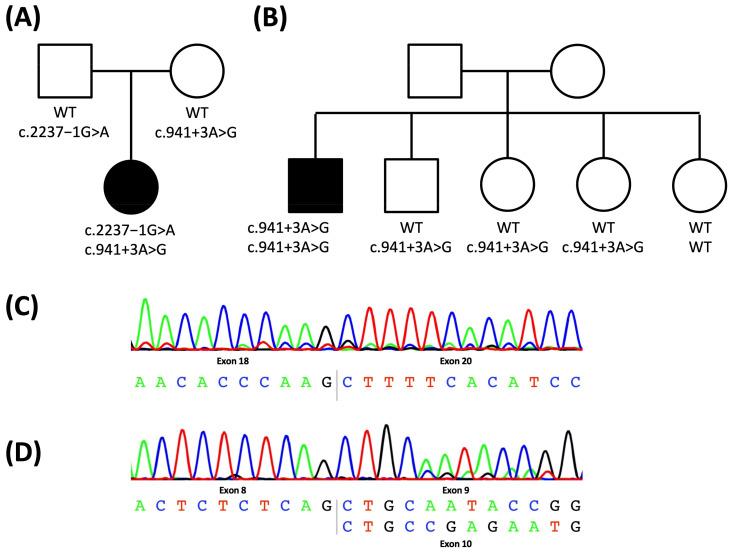
Pedigree of the family of patient 1 (**A**) and patient 2 (**B**): the mutational status is indicated as genotype of the two alleles; cDNA sequence of P1: complete skipping of exon 19 due to the c.2237−1G > A variant (**C**) and partial skipping of exon 9 due to the c.941 + 3A > G variant (**D**). WT: wildtype, square: male, circle: female, white: unaffected, black: affected.

An extensive metabolic workup did not reveal any disease-causing abnormality. Subsequently, HSP-*SPAST* (SPG4), HSP/ATX-*SPG7* (SPG7) as well as ATX-*ATXN1* (SCA1), ATX-*ATXN2* (SCA2), ATX-*ATXN3* (SCA3), ATX-*CACNA1A* (SCA6), ATX/HSP-*SACS* (ARSACS), and Alexander’s disease (*GFAP*) were excluded. In early 2018, a patient-parent trio exome sequencing did not reveal any disease-causing variants. However, re-evaluating exome data in 2020 within the GPAP platform of the SolveRD project, identified two potentially pathogenic, intronic variants in the *VPS13D* gene (NM_018156.4: c.941 + 3A > G and c.2237−1G > A), which had been linked to spastic ataxia and spastic paraplegia as recently as in mid-2018 (Figure 2A).

The clinical presentation with spastic ataxia was in line with previously described cases (see Table 1). However, tremor has previously only been described in two patients [7].

### 2.2. Case Report: Patient 2

The second patient, who is now 68 years old, developed the first symptoms at the age of 45 years. He developed progressing walking difficulties, resulting in the inability to run and had to give up playing golf as a hobby. He presented with stiffness, difficulty in walking and running leading to recurrent falls. On last examination, age 68 years old, he had bilateral spastic paraplegia in the lower limbs associated with pyramidal weakness, brisk reflexes and upward plantars. This was associated with neurogenic bladder and bowel. He is able to mobilize independently and uses functional electrical stimulator. The rest of the neurological examination was unremarkable. His Barthel score was 85/100.

Diagnostic work up including cMRI and muscle biopsy were normal.

He has one unaffected brother and 3 unaffected sisters. The parents are healthy as well and there is no family history for hereditary spastic paraplegia. Exome sequencing in 2018 did not reveal any disease causing variants. However, a re-evaluation of the exome data in 2020, identified a potentially pathogenic, homozygous intronic variant in the *VPS13D* gene outside the canonical splice site (NM_018156.4: c.941 + 3A > G; Figure 2B). The variant was previously reported in the compound heterozygous state but without proof of an effect on splicing. The clinical presentation with late-onset spastic paraplegia in *VPS13D* mutation carriers has previously been described (see Table 1).

### 2.3. Characterization of the Intronic Variants for a Splicing Effect

In patient P1, two intronic variants, a maternal c.941 + 3A > G (NM_018156.4) and a paternal c.2237−1G > A (NM_018156.4) variant, were detected in *VPS13D* in the compound heterozygous state (Figure 2A). According to the ACMG guidelines, both variants are classified as likely pathogenic.

P2 was identified as homozygous for the c.941 + 3A > G variant. Segregation in the family showed the brother and two of the sisters to be heterozygous for the variant and the third sister not to carry any mutated allele (Figure 2B).

Variants at both positions have been described previously [6] in combination with other variants; however, no confirmation of the splice site effect of either variant was performed.

Sequencing of cDNA from blood and fibroblast cultures of P1 as well as her parents showed a complete skipping of exon 19 due to the c.2237-1G > A variant, which lies within the canonical splice site just before exon 19 (Figure 2C). Of note, the c.941 + 3A > G variant, located outside the canonical splice site region at the beginning of Intron 9, resulted in only partial skipping of exon 9 (Figure 2D).

**Figure 3:**
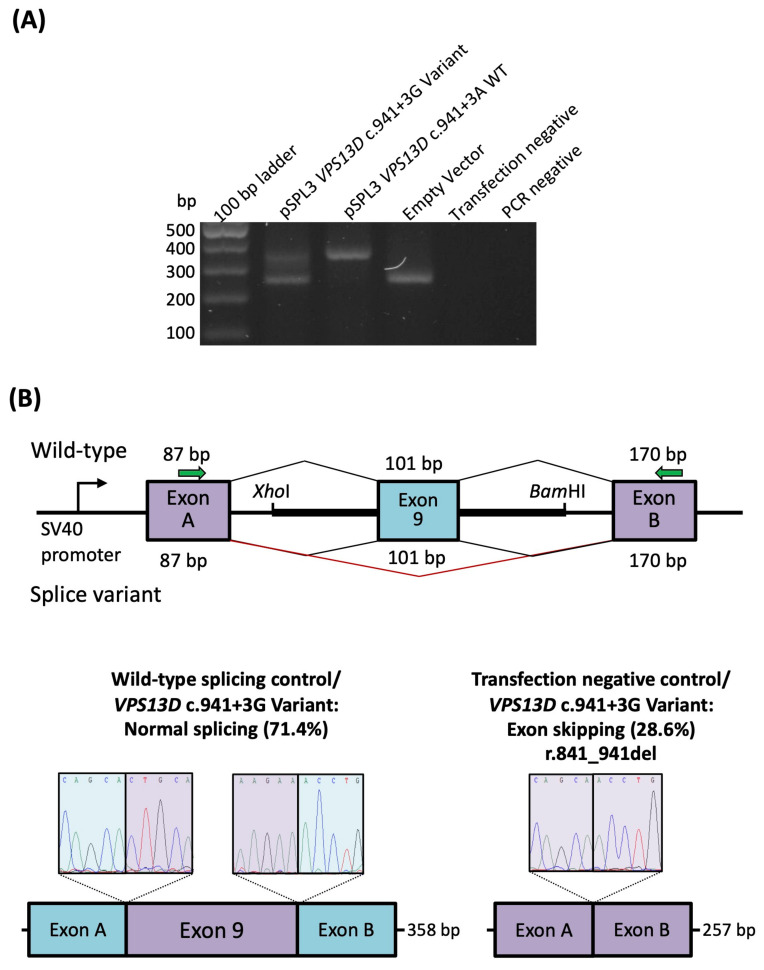
RNA analysis of the *VPS13D* c.941 + 3A > G variant. (**A**) Gel electrophoresis of the RT-PCR of RNA-transcribed cDNA from HEK 293T cells with the *VPS13D* c.941 + 3G variant, wild-type c.941 + 3A, and empty pSPL3 vector amplicons. Transfection-negative and PCR-negative controls performed as expected. (**B**) The vector construct of the in vitro splice assay illustrates the wild-type (upper splice profile) or mutant (lower splice profile) amplicons inserted between exons A and B of the pSPL3 vector with splicing schematic of wild-type and mutant shown below. The c.941 + 3A > G variant causes leaky splicing that results in either wild-type splicing (left sequencing panel, observed in 5 out of 7 (71.4%) amplicons) or a skipping of exon 9, resulting in a deletion of 101 bp (r.841_941del, observed in 2 out of 7 (28.6%) amplicons) that appears the same as the transfection negative control (right sequencing panel). WT: wild-type.

In addition to the sequencing of cDNA derived from blood and fibroblasts of patient P1, we also performed a Minigene assay with TA cloning to test for an effect of the c.941 + 3A > G variant and approximate the abundance of the two transcript forms. There was evidence of leaky splicing (Figure 3A) with 5/7 (~70%) of the transcripts representing the wild-type including all exons, and 2/7 (~30%) of the transcripts corresponding to an out of-frame transcript with skipping of exon 9 (r.841_941del) (Figure 3B) confirming the patient P1-derived data.

### 2.4. Mitochondrial Network Analysis

**Figure 4:**
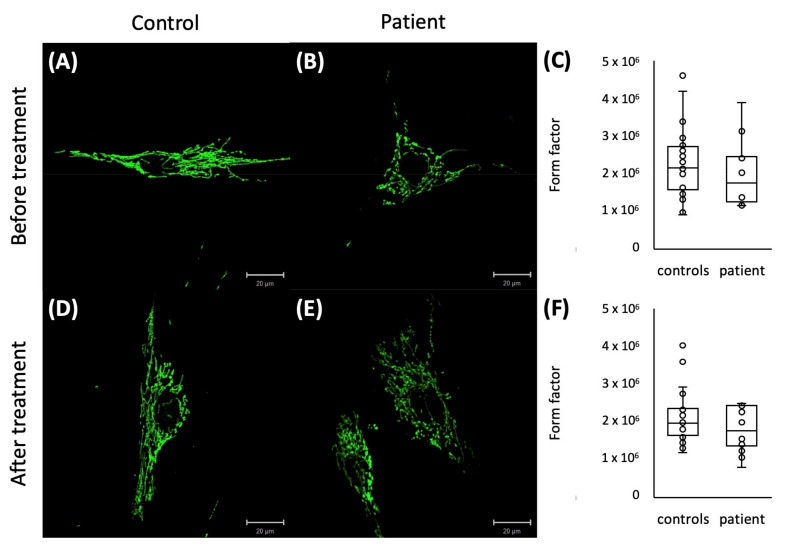
Representative pictures of immunostaining of cultured fibroblasts with GRP75 of a control (**A**,**D**) and a patient (**B**,**E**) before (**A**,**B**) and after paraquat treatment (**D**,**E**) with a trend to a reduced mitochondrial branching in P1 before (**C**) and after paraquat treatment (**F**) represented by a reduced form factor.

Since disturbance of the mitochondrial network has been reported in other *VPS13D* patients [6], we also stained the mitochondrial network in available cultured fibroblasts from patient P1. The form factor analysis showed a slightly reduced degree of mitochondrial branching in the patient’s fibroblasts compared to the fibroblast of healthy controls before and after treatment with paraquat (Figure 4).

## 3. Discussion

In this study, we diagnosed two patients with *VPS13D*-related disorder based on the identification of pathogenic, biallelic, and intronic variants. We were able to show a skipping of exon 19 due to the c.2237-1G > A variant and a partial skipping of exon 9 in approximately 30 % of the transcripts due to the c.941 + 3A > G. In addition, as previously reported [6], the mitochondrial network was impaired in patient-derived fibroblasts.

Interestingly, this is also the first report of DBS in a patient with *VPS13D*-related disorder. One of our patients (P1) had a debilitating Holmes-like tremor. All components of the tremor were reduced by VIM DBS to the effect that she regained independence in daily life including eating and drinking. Of note, the attenuation of voice tremor improved communication. The VIM is a common target in tremor syndromes with essential tremor being the most frequent tremor syndrome [13]. There are also favorable reports of more than 30 patients with Holmes tremor treated with VIM DBS [14,15], but in some cases, DBS effects were transient [14]. On the other hand, there are also reports of cerebellar DBS improving both tremor and ataxia; however, only a few reports are available in the literature [16]. Since tremor seems to be a less frequent symptom in *VPS13D*-related disorder with only 3 reported patients (<10%), the usefulness of DBS as a treatment option in *VPS13D*-related disorder is unclear.

The phenotypic spectrum of *VPS13D*-related disorder appears to be broad with some patients suffering from spastic ataxia, others from spastic paraplegia or more complex disorders with additional symptoms such as tremor, dystonia, myoclonus, or intellectual disability being common. Additionally, oculomotor dysfunction in form of saccadic intrusion [6,17] as well as Leigh-like leukoencephalopathy [7], as present in P1, have likewise been described. While there seems to be no clear genotype-phenotype relation, patients carrying biallelic missense variants [8,12] or non-canonical splice site variants such as P2 seem to have a later age at onset (mean: 30 years) and a less severe phenotype than patients carrying a truncating variant, including canonical splice site variants, in combination with a milder missense or non-canonical splice site change [6,7,8,9,10] such as P1 (mean age at onset: 14 years). However, there are also cases reported who do not fit this pattern [7,11,12]. For example, two patients with a biallelic missense variant developed gait problems and global developmental delay, respectively, before the age of 2 years [7], while another patient with a truncating and a missense variant developed mild gait difficulties after the age of 30 years [11]. For another two patients with biallelic missense variants, minimal data are available with the only information having an age at onset of 6 and 13 years, respectively [12].

Another novel, remarkable feature in these two patients is the presence of intronic variants on both alleles. We were able to show an effect on the splicing of both variants. Especially the partial splice site effect of the c.941 + 3A > G variant and as a result the pathogenicity of this variant is important to consider, since non-canonical splice site variants can often be overlooked due to the filtering parameters, which was the case in both patients described here. It is common to select functional annotation criteria to filter variants for their likelihood to affect the protein level, e.g., using the classifications high and moderate, and excluding low and modifier [18]. According to annotation criteria, the c.941 + 3A > G variant was considered not harmful on the protein level and classified as low. Searching for high and moderate variants in our patient did not reveal the disease’s cause. Only by broadening the search parameter the diagnosis of a *VPS13D*-related disorder was made. Therefore, also including variants classified as low can be useful, especially in cases of suspected recessive disorder and identification of only a single variant as was the case in P1.

## 4. Materials and Methods

### 4.1. Identification of Variants and Additional Patient

Previously generated exome sequencing data (Agilent SureSelect Human All Exon V6 (Illumina, Inc., San Diego, CA, USA) at Centogene (Rostock, Germany) of the index patient P1 and her parents were included in GPAP (Genome-Phenome Analysis Platform; https://platform.rd-connect.eu; accessed 2 September 2021) [19] of the SolveRD project (https://solve-rd.eu/; accessed 2 September 2021) [20,21]. Re-analysis of the exome data was performed by filtering variants for inheritance (de novo, recessive or autosomal dominant with reduced penetrance), frequency in the population by gnomAD [22] allele frequency (de novo: <0.001, recessive: <0.01) and variant type according to SnpEff [18] (high or moderate). Different gene lists, including the ERN- RND gene lists (European Reference Network for rare neurological disorders), were used to limit the number of genes [21]. After identifying a splice site variant in *VPS13D* inherited from the father, variant type “low” according to SnpEff was included to search for a second variant in *VPS13D* inherited from the mother. Variants were classified according to ACMG guidelines using VarSome on 2 September 2021 [23].

Prompted by the identification of biallelic variants in *VPS13D* in P1, we searched the GPAP platform for additional cases with two *VPS13D* variants following the same filtering criteria as mentioned above as well as searching for both variants found in P1. As a result, the submitter of another patient with two variants and a phenotype including ataxia, tremor and/or spasticity were contacted via the GPAP correspondence tool.

### 4.2. Sanger Sequencing and Sequencing of cDNA

Blood and fibroblasts cultured from skin biopsy were acquired from P1 as well as both parents. RNA from blood (PAX tube system) and cultured fibroblasts was extracted using QIAmp RNA Extraction Kit (Qiagen, Hilden, Germany). Subsequently, the complementary DNA (cDNA) was synthesized by reverse transcriptase using Oligo-dT-Nucleotides of the Maxima First Strand cDNA Synthesis Kit (ThermoFisher, Waltham, MA, USA) as primers. PCR was conducted (VPS13D_c.941+3_F: TGAAGTCAAAGCTGGCTTCATT; VPS13D_c.941+3_R: CTGACAGTCCCTCAACCACA; VPS13D_c.2237-1_F: ATGCAGCTCGAGTTTTCAGATGT; VPS13D_c.2237-1_R: GAGCACAGAAATGTACCGGC) and the products Sanger sequenced.

In P2, as well as in three sisters and one brother, Sanger sequencing was performed but no blood or cell lines for RNA extraction were available.

### 4.3. Quantification of Exon Skipping

RNA studies to assay the effect of the splice region variant c.941 + 3A > G were performed as previously described [24,25]. Briefly, exon 9 (101 bp), as well as flanking intronic 5′ (126 bp) and 3′ (139 bp) sequences were directly PCR-amplified from a control individual and the proband with primers containing an additional *Xho*I and *Bam*HI restriction site (forward primer with *Xho*I restriction site: 5′-aattctcgagCAGTTTGCACTGGGTTTCAA-3′ and reverse primer with *Bam*HI restriction site: 5′- attggatccGCATTCCAAGGACCAGAAA-3′). After PCR amplification and clean-up, restriction enzyme digestion of the PCR fragment and the pSPL3 exon trapping vector was performed prior to ligation between exon A and exon B of the linearized pSPL3-vector. The vector was transformed into DH5α competent cells (NEB 5-alpha, New England Biolabs, Frankfurt, Germany) and plated and incubated overnight. The wild-type and mutant-containing vector sequences were confirmed by Sanger sequence.

Vectors containing the mutant or wild-type sequence were transfected into HEK 293T cells (ATCC, Manassas, VA, USA) at a density of 2 × 10^5^ cells per mL. 1 µg of the respective pSPL3 vectors was transiently transfected using 3 µL of FuGENE 6 Transfection Reagent (Promega, Walldorf, Germany). An empty vector and transfection negative reactions were included as controls. The transfected cells were harvested 24 h after transfection. Total RNA was prepared using miRNAeasy Mini Kit (Qiagen, Hilden, Germany). Approximately 1 µg of RNA was reverse transcribed using the High Capacity RNA-to-cDNA Kit (Applied Biosystems, Waltham, MA, USA) following the manufacturer’s protocols. The cDNA was PCR amplified using vector-specific SD6 forward (5′-TCTGAGTCACCTGGACAACC-3′) and SA2 reverse (5′-ATCTCAGTGGTATTTGTGAGC-3′) primers. The amplified fragments were visualized on a 1% agarose gel and Sanger sequenced. cDNA amplicons containing the variant were TA cloned into the pCR2.1 vector (ThermoFisher, Darmstadt, Germany) following standard protocols. Seven colonies were cultured in LB-ampicillin medium. Plasmids were extracted and Sanger sequenced using M13 primers.

### 4.4. Form Factor of the Mitochondrial Network

Fibroblast cell lines were established for P1 and three controls and cultured in medium (Dulbecco’s modified Eagle’s medium (DMEM, Thermo Scientific, Waltham, MA, USA), 10% fetal bovine serum (Life Technologies), 1% penicillin/streptomycin (Life Technologies)) at 37 °C and 5% CO_2_ in a humidified atmosphere. The form factor was determined as previously described [26,27]. Cells were either stained directly or after treatment with paraquat for 5 h. After staining the mitochondrial network in fibroblasts with an anti-GRP75 antibody (1:1000, Abcam, Cambridge, MA, USA) in combination with the Zenon immunolabelling kit (Invitrogen, Carlsbad, CA, USA) according to the manufacturer’s protocol, mitochondria area and outline were measured based on single cell images.

## 5. Conclusions

In conclusion, we have described two patients with pathogenic, biallelic intronic *VPS13D* variants and consequently *VPS13D*-related disorder manifesting as spastic ataxia with severe tremor syndrome and as pure spastic paraplegia, respectively. Furthermore, we present the first case of successful VIM DBS for severe tremor in a patient with a *VPS13D*-related disorder.

## Figures and Tables

**Table 1:** Summary of clinical feature of both patients P1 and P2 as well as reported patients in the literature.

Category	P1	P2	Literature (*n* = 31 [5,6,7,8,9,10,11]) ^1^
Sex	Female	Male	male 14/29
Origin	Germany	UK	Europe: 18/31
			Asia: 10/31
			Other: 3/31
Age of Onset	6 years	45 years	Mean: 20 years (range: 0–63 years)
Age at Examination	31 years	47 years	Mean: 35 years (range: 2–72 years)
First signs and symptom	Gait disturbance (unsteady gait)	Gait disturbance (unable to run)	Gait disturbance: 18/26 Developmental delay: 3/26 Tremor: 2/26 Other ^2^: 3/26
Ataxia Upper; Lower Limb	yes; yes	yes; yes	21/24 10/14; 13/15
Spasticity Upper; Lower Limb Babinski Sign	yes; yes yes	yes; yes yes	9/13 1/8; 9/13 22/27
Hyperreflexia Upper; Lower Limb	yes; yes	yes; yes	24/27 8/12; 24/27
Paresis Upper; Lower Limb	no; yes	yes; yes	8/12 4/7; 8/12
Ambulation	Wheelchair user	Balance issues	Wheelchair: 6/13 Walker: 2/13 Independent: 5/13
Tremor	yes	no	2/4
Oculomotoric Square Waves Jerks Nystagmus Suppresion of VOR	yes yes pathological	no no normal	7/10 0/2 0/1
Dysarthria	yes	no	10/13
MRI abnormalities	Leukencephalopathy	none	Normal: 6/20 Cerebellar atrophy: 11/20 Leukencephalopathie: 3/20
Response to treatment	Levodopa (mild), VIM- DBS (good)	Poor to Baclofen	n. a.

^1^ not all symptoms were reported in all patients. Number of patient with information on specific symptoms is indicated behind the slash. ^2^ intellectual disability, chorioretinal dystrophy, and hypotonia.

## Data Availability

The raw data supporting the conclusions of this article will be made available by the authors on a reasonable request.

## Supplementary Materials

The following supporting information can be downloaded at: http://doi.org/10.5281/zenodo.7378856, Video S1: P1 before deep brain stimulation, Video S2: P1 after deep brain stimulation.
